# The associations of comorbidities and consumption of fruit and vegetable with quality of life among stomach cancer survivors

**DOI:** 10.1186/s12955-018-0886-y

**Published:** 2018-04-12

**Authors:** Ji-Wei Wang, Cheng-Gang Zhang, Qing-Long Deng, Wan-Li Chen, Xian Wang, Jin-Ming Yu

**Affiliations:** 1 Key Lab of Health Technology Assessment of National Health Commission and Key Lab of Public Health Safety of Ministry of Education, School of Public Health, Fudan University, 130 Dong-An Road, Shanghai, 200032 China; 2 Shanghai Xuhui Center for Disease Control and Prevention, 50 Young-Chuan Road, Shanghai, 200237 China

**Keywords:** Stomach cancer survivors, Quality of life, QLQ-C30, Comorbidity, Consumption of fruit and vegetable

## Abstract

**Background:**

Stomach cancer survivors (SCS) often carry the dual burden of the cancer itself and other comorbidities; meanwhile, they are highly motivated to seek health advice about lifestyles to improve their health and quality of life (QOL). The associations of the comorbidity and the consumption of vegetable and fruit with QOL remain even less clear among the SCS. This study aimed to investigate the associations of comorbidities and consumption of fruit and vegetable with QOL among SCS.

**Methods:**

A cross-sectional study was conducted among 969 SCS between April and July 2015 in Shanghai, People’s Republic of China. Data were collected using a self-reported questionnaire, which included questions on sociodemographic characteristics, comorbidities and fruit and vegetable consumption, and a simplified Chinese version of the European Organization for Research and Treatment quality of life version 3 (EORTC QLQ-C30) questionnaire. In order to mitigate the bias caused by confounding factors, multiple linear regression models were employed to calculate the adjusted means of QOL scores.

**Results:**

The proportion of participants without any comorbidity was only 23.3%, and the most common comorbidity among SCS was digestive diseases (49.8%). Participants with comorbidity generally reported lower scores for global health and functioning subscales and higher scores for symptom in EORTC QLQ-C30 compared to participants without comorbidity, indicating poorer QOL. Higher scores in most functioning subscales and lower scores in some symptoms subscales were found in participants (38.7%) who ate more than 250 g vegetables every day, compared to participants with less vegetable consumption, and in participants (58.1%) who ate fruit every day, compared to participants who didn’t eat fruit every day indicating better QOL.

**Conclusions:**

The comorbidities are common health problems among SCS and have significantly negative influence on QOL, and participants with comorbidities generally reported lower QOL scores. The enough vegetables and fruit consumption are positively associated with QOL of SCS. These findings suggested that a multidisciplinary team approach and a variety of delivery systems are needed to address the medical, psychosocial, and lifestyle components for enriching patient-centered care among SCS.

## Background

The stomach cancer incidence has been decreasing substantially in most parts of the world over the recent few decades [1–3]. However, there was still almost one million new cases of stomach cancer were estimated to have occurred in 2012, more than 70% of cases occurred in developing countries, and half of cases occured in Eastern Asia (mainly in China) [1]. Meanwhile, the number of stomach cancer survivors (SCS) in China may increase over time as a result of early detection and improved treatment [4].

Traditional indicators, such as survival and cure rate, cannot sufficiently reflect the effect of treatments and the health conditions of SCS. Quality of Life (QOL) is increasingly being used as a primary outcome measure in studies to evaluate the effectiveness of treatment, and long-term survival and functional status of cancer survivors, which means the individual’s experience about the survival-related goals, expectations, standards and concerns in different cultures and value systems [5]. QOL is a multidimensional concept which covers various survival aspects related to physical, emotional, mental, sexual or social functioning [6]. World Health Organization emphasized the importance of the improvement of QOL in their global cancer control strategy [7].

Previous studies indicated that the QOL of SCS could be undermined by the side effects of cancer and its treatments, such as physiological effect of nausea or problems with swallowing [8], and a psychological effect of depression [9], and a social effect withdrawal due to embarrassment about being ill [10, 11]. Assessment of QOL to monitor and care long-term SCS about medical and psychologic consequences of their cancer treatment and rehabilitation is imperative.

Cancer patients generally report more comorbidity than do patients without a history of cancer, with prevalence varying between cancer sites [12–14]. The number and severity of a survivor’s comorbidities also affect clinical care, treatment options, health service needs, and prognosis of cancer [15, 16]. There are a few studies which specifically investigated the association of comorbidity with the QOL of cancer survivors, and indicated that cancer survivors with certain comorbidities such as cardiovascular, respiratory, digestive, and musculoskeletal diseases had lower QOL [17, 18]. However, the investigation of prevalence of comorbidity and the associations of comorbidities with QOL among SCS were still scarce.

Lifestyle factors, in terms of diet, have been shown to be important predictors of occurrence and recurrence cancer, as well as the QOL of cancer survivors. A high consumption of vegetables and fruit is strongly and consistently associated with a reduced stomach cancer risk [19–21]. Persons with a high intake of vegetables and fruit tended to have a 50% reduction in stomach cancer risk, compared to the ones with a low intake [22]. In addition, Brown et al. observed that post-diagnosis diet quality was directly associated with subsequent mental and physical functioning in the breast cancer survivors, and this association was stronger for mental functioning than for physical functioning [23]. The breast cancer survivor with better nutritional status had better functioning scales (physical, role, cognitive, social) and QOL, and experienced fewer clinical symptoms [24]. However, few studies have assessed the impact of fruit and vegetable intake on QOL among SCS. It is important to provide more information to SCS to help them to learn the association of consumption of fruit and vegetable with QOL.

The specific objectives of this study are to investigate the prevalence of comorbidity and consumption of fruit and vegetable, and the associations of comorbidities and consumption of fruit and vegetable with QOL among SCS. A better comprehensive understanding of the influence of comorbidities and consumption of fruit and vegetable on QOL post-diagnosis is needed to improve model of care and maximizes QOL among SCS.

## Methods

### Recruitments

Between April and June in 2015, participants were recruited from seventeen multi-community cancer rehabilitation centers, all of which were affiliated Shanghai Cancer Rehabilitation Club (SCRC), Shanghai, China, a non-government organization serving cancer survivors exclusively. It covered all the 17 districts in Shanghai and has about 13,500 cancer survivor members in Shanghai by the end of 2014. This self-help support group aiming at improving survivors’ quality of life regularly offers rehabilitation activities including physical exercise, relaxation training, nutritional and dietary counseling, psychotherapy and a variety of leisure aactivities [25]. The criteria for study enrollment are at least 16 years of age, stomach cancer as the first primary cancer, completed active treatment, able to independently participate in the activities of cancer rehabilitation club, and no cognitive impairment.

Total 1354 SCS have registered in the SCRC by the end of 2014 meet the above inclusion criteria. All members of the SCRC who intended to continue to participate in the SCRC were required to register annually, including both former and new members. The survey invitations were sent through short text messages or/and phone calls to all 1354 SCS. Among all these SCS, 131 were unable to reach because of deaths, migration or refusing to response; 254 failed to participate the survey either because they had no time, or because their health status or literacy ability was too poor. Finally, 969 (71.57%) participated this survey and formed our final sample, and completed the self-administered questionnaire, in his/her own home, or community activity center affiliated SCRC, which normally took 40–60 min.

### Instruments

#### Demographic characteristics

The demographic characteristics included age, gender, body mass index, years since diagnosis, marital status, the highest educational level achieved, and monthly household income per capita.

#### Comorbidities

Participants were asked to indicate either “yes” or “no” on a list of comorbidities including hypertension, diabetes mellitus, heart diseases, stroke, respiratory diseases, digestive diseases, musculoskeletal diseases.

### Consumption of fruit and vegetable

Vegetables intake was obtained using the choice question “How much vegetables do you eat?”. The options were “≤50 g/day”, “>50g/day and ≤100g/day”, “>100g/day and ≤150g/day”, “>150g/day and ≤250g/day”, “>250g/day and ≤500g/day” and “> 500 g/day”. Fruit intake was obtained using choice question “Do you eat fruit?”. The options were “No”, “1 time/week”, “2 times/week”, “3 times/week”, “4–5 times/week”, “Everyday”.

### QOL measurements

The QOL was measured by the European Organization for Research and Treatment quality of life version 3 questionnaire (EORTC QLQ-C30) simplified Chinese version [26]. The EORTC QLQ-C30 core questionnaire contains five functional scales (physical, role, cognitive, emotional, and social), three symptoms scales (fatigue, pain, and nausea and vomiting), a global health scale, a number of single items assessing additional symptoms commonly reported by cancer patients (dyspnea, appetite loss, insomnia, constipation, and diarrhea) and financial impact of the disease. For most items, four response categories from 1 (not at all) to 4 (very much) are employed; two items (overall health, overall quality of life) have response categories ranging from 1 to 7. The scoring systems are organized such that a high scale score represents a higher response level. Thus a high score for a functional scale, the global health status or overall QOL represents a high or healthy status or a high QOL; however, a high score for the symptom scales represents a high level of symptomatology or problems. The scoring of the EORTC QLQ-C30 items was performed according to the EORTC scoring manual [27].

### Statistical analysis

Standard statistical methods were used to examine potential differences in covariates by one-way analysis of variance (ANOVA) for continuous variables followed by Bonferroni post hoc tests. Comorbidity, vegetable intake, and fruit intake were conducted in the statistical analyses as dichotomous variables (yes/no, ≥250 g/day /< 250 g/day, and everyday/not every day, respectively). Means and standard deviations were calculated for continuous variables, and numbers and percentages were computed for categorical variables. Multiple linear regression models were used to compute regression coefficients (β) and associated 95% confidence intervals as estimates of the mean difference of QOL scores associated with the presence or absence of comorbidities, adjusting for potential confounding variables. The following potentially confounding variables were included in all regression models: age (continuous), Body Mass Index (continuous), years since diagnosis (continuous), household income (continuous), education (junior high school or less, senior high school or vocational school, and junior college or above), and current marital status (married/living with partner or divorced/widowed/ separated/single). Linear regression was applied to each of the variables measured in EORTC QLQ-C30. Comorbidity and other diseases (hypertension, diabetes mellitus, etc.) were not included in one model. Statistical tests were based on a two-tailed probability with a significance level of 0.05. Statistical analyses were performed using the Statistical Package for the Social Sciences (SPSS) for Windows (Version 21.0).

## Results

### The characteristics of the study sample

The characteristics of the participants are shown in Table 1. Forty-four percent of the sample was 60 years old or above. Majority of them were married (89.2%) and had at least one comorbidity (76.7%). 15.1, 63.8 and 21.1% of the participants were underweight, normal weight and overweight or obese, respectively. In terms of the types of comorbidity, the proportion of SCS who suffered from digestive diseases (49.8%) was the highest. Other comorbidities are listed according to their prevalence as follows: hypertension (30.4%), diabetes mellitus (12.8%), heart diseases (22.9%), stroke (6.7), respiratory diseases (12.3%), musculoskeletal diseases (29.6%). 38.7% of the participants ate more than 250 g vegetables every day, and 58.1% of participants who ate fruit every day.

**Table 1:** Summary statistics of 969 stomach cancer survivors

variables	Number(%)
Sex	
Male	515(53.1)
Female	454(46.9)
Age(years):	
≤ 59	309(31.9)
60~ 69	426(44.0)
≥ 70	234(24.1)
Body mass index (BMI, kg/m2)	
< 18.5	146(15.1)
18.5~ 24	618(63.8)
25~ 29	198(20.4)
≥ 30	7(0.7)
Years since diagnosis	
< 2	90(9.3)
2~ 5	229(23.6)
> 5	650(67.1)
Marital status	
Married/with partner	864(89.2)
Divorced/widowed/separated/single	105(10.8)
Education	
Junior high school or less	457(47.2)
Senior high school or vocational school	342(35.3)
Junior college or above	170(17.5)
Monthly household income per capita (¥)	
< 2000	257(26.5)
2000~ 4000	530(54.7)
> 4000	182(18.8)
Number of comorbidities	
0	226(23.3)
1	250(25.8)
2	193(19.9)
≥ 3	300(31.0)
Comorbidities	
Hypertension	295(30.4)
Diabetes	124(12.8)
Cardiovascular	222(22.9)
Stroke	65(6.7)
Respiratory diseases	119(12.3)
Digestive diseases	483(49.8)
Musculoskeletal diseases	287(29.6)
Vegetable intake	
< 250 g/day	594(61.3)
≥ 250 g/day	375(38.7)
Fruit intake everyday	
Yes	563(58.1)
No	406(41.9)

### The association between comorbidities and adjusted scores of QOL

The association between comorbidities and adjusted scores of QOL is presented in Table 2. The participants with self-reported CCD generally reported lower scores for most EORTC QLQ-C30 scales when compared to subjects without these CCD, indicating poorer QOL. The influences of diabetes, heart diseases, stroke, respiratory diseases and musculoskeletal illnesses or digestive diseases on EORTC QLQC30 scores were of a similar magnitude and were larger than the influence of hypertension.

**Table 2:** The association between comorbidities and adjusted scores of QOL among stomach cancer survivors

	With Comorbidity, (without comorbidity)	With HPT, (without HPT)	With DM, (without DM)	With HD, (without HD)	With STR (without STR)	With RD (without RD)	With DD (without DD)	With MD (without MD)
PF	− 3.52***	−1.72***	−2.79***	−4.85***	−6.87***	−5.77***	− 2.54***	− 4.73***
	(85.94)	(83.26)	(83.24)	(83.86)	(83.01)	(83.48)	(83.66)	(84.16)
RF	−2.67***	−1.15*	−3.13***	−4.30***	− 6.42***	−5.90***	− 1.63***	− 4.38***
	(91.89)	(90.19)	(90.36)	(90.92)	(90.31)	(91.02)	(91.48)	(91.97)
EF	−3.75***	−1.43***	− 3.00***	−4.98***	−5.84***	− 4.93***	− 3.28***	− 5.31***
	(85.81)	(85.53)	(85.82)	(86.49)	(85.61)	(85.78)	(86.70)	(87.01)
CF	−4.45***	−1.04*	−3.17***	−5.92***	−6.64***	−6.14***	−3.86***	− 6.45***
	(83.18)	(79.37)	(79.24)	(80.45)	(78.97)	(79.87)	(81.35)	(81.46)
SF	−4.72***	−1.59**	−2.70***	−6.26***	−8.21***	−8.80***	− 4.29***	−5.76***
	(81.51)	(77.33)	(77.28)	(78.60)	(77.27)	(77.87)	(79.33)	(79.00)
QL	−6.03***	−3.18***	−4.63***	−7.21***	− 8.13***	−7.26***	− 4.28***	−6.37***
	(68.41)	(64.04)	(63.66)	(64.87)	(63.25)	(64.03)	(64.98)	(65.85)
FA	7.37***	1.89***	3.56***	6.85***	9.12***	7.55***	6.07***	7.38***
	(23.48)	(28.18)	(28.24)	(27.09)	(28.34)	(27.80)	(25.39)	(26.14)
NV	0.41	0.42	0.84*	2.26***	2.77***	3.29***	0.50*	1.60***
	(4.72)	(5.75)	(5.56)	(5.05)	(5.29)	(5.11)	(4.80)	(4.97)
PA	6.41***	2.09***	3.15***	6.66***	7.74***	7.12***	5.04***	12.00***
	(10.96)	(16.09)	(16.17)	(15.24)	(16.41)	(15.79)	(13.40)	(12.79)
DY	5.53***	2.01***	2.87***	7.93***	8.18***	13.88***	3.83***	5.23***
	(12.48)	(14.49)	(14.97)	(13.18)	(14.95)	(13.94)	(13.99)	(13.93)
SL	4.03***	0.70	2.17***	4.30***	5.43***	5.64***	3.18***	3.50***
	(13.42)	(18.22)	(18.27)	(16.99)	(18.26)	(17.74)	(15.83)	(16.32)
AP	1.81***	0.74	2.02***	4.19***	5.00***	5.78***	2.07***	3.10***
	(10.45)	(12.15)	(12.68)	(11.52)	(12.45)	(11.90)	(10.85)	(11.56)
CO	4.32***	−0.17	2.62***	4.00***	4.61***	3.27***	4.19***	3.84***
	(8.45)	(11.47)	(11.73)	(11.22)	(11.34)	(11.39)	(8.72)	(10.14)
DI	2.68***	1.11**	1.53**	2.60***	2.66***	4.29***	3.12***	2.99***
	(9.37)	(11.75)	(11.49)	(10.82)	(11.53)	(11.06)	(8.86)	(10.09)
FI	5.58***	1.81*	2.19*	6.47***	7.01***	8.68***	5.07***	6.33***
	(27.96)	(32.01)	(31.76)	(30.40)	(31.45)	(31.04)	(28.69)	(29.67)

Abbreviations: *AP* Appetite loss, *BMI* Body mass index, *CF* Cognitive functioning, *CO* Constipation, *DD* Digestive diseases, *DI* Diarrhea, *DM* Diabetes mellitus, *DY* Dyspnea; emotional functioning, *EORTC* *QLQ-C30* European Organization for Research and Treatment quality of life version 3 questionnaire, *FA* Fatigue, *FI* financial difficulties, *HD* Heart diseases, *HPT* Hypertension, *MD* Musculoskeletal diseases, *NV* Nausea and vomiting, *PA* Pain, *PF* Physical functioning, *QL* Global health, *QOL* Quality of life, *RD* Respiratory diseases, *RF* Role functioning, *SF* Social functioning, *SL* Insomnia, *SCS* Stomach cancer survivors; stroke, STR

Notes: *P,0.05, **P,0.01, ***P,0.001

Multiple linear regression, adjusted for influence of sex, age, marital status, BMI, education, household income, time after diagnosis

### The association between consumption of fruit and vegetable and adjusted scores of QOL

The association between consumption of fruit and vegetable and adjusted scores of QOL is presented in Table 3. The participants who ate more than 250 g vegetables every day, and participants who ate fruit every day, reported higher scores in most functioning subscales and lower scores in some symptoms subscales than participants who ate equal or less than 250 g vegetables every day, and participants who didn’t eat fruit every day, respectively, indicating better QOL.

**Table 3:** The association between consumption of fruit and vegetable and adjusted scores of QOL

	Vegetable intake	Fruit intake everyday
	≥250 g/day (< 250 g/day)	Yes (No)
PF	1.52***(82.20)	0.28(80.98)
RF	1.01*(89.59)	1.54***(87.47)
EF	1.00**(84.54)	1.12**(83.49)
CF	1.68***(77.58)	1.60***(75.31)
SF	1.01*(75.74)	0.88(74.58)
QL	2.15***(61.33)	2.00***(59.70)
FA	−1.17**(29.99)	−1.01*(31.58)
NV	−0.54*(6.70)	−0.27(6.60)
PA	−0.48(17.49)	− 1.10*(18.44)
DY	−1.47**(16.26)	− 0.57(17.70)
SL	0.53(19.27)	0.46(21.70)
AP	−1.52***(14.43)	−1.56***(15.12)
CO	−1.12*(13.24)	−1.02*(13.10)
DI	−0.74*(12.17)	−0.33(12.28)
FI	−0.85(32.01)	−1.91**(34.18)

Abbreviations: *AP* Appetite loss, *BMI* Body mass index, *CF* Cognitive functioning, *CO* Constipation, *DI* Diarrhea, *DY* Dyspnea; emotional functioning, *EORTC* *QLQ-C30* European Organization for Research and Treatment quality of life version 3 questionnaire, *FA* Fatigue, *FI* Financial difficulties, *NV* Nausea and vomiting, *PA* Pain, *PF* Physical functioning, *QL* Global health, *QOL* Quality of life, *RF* Role functioning, *SF* Social functioning, *SL* Insomnia, *SCS* Stomach cancer survivors; stroke, STR

Notes: *P,0.05, **P,0.01, ***P,0.001

Multiple linear regression adjusted for influence of sex, age, marital status, BMI, education, household income, time after diagnosis,number of comorbidities

## Discussion

This study examined the associations of comorbidities and consumption of fruit and vegetable with QOL among SCS. The SCS with comorbidities had a lower QOL score compared to the SCS without comorbidities, and the SCS with more consumption of fruit and vegetable had a higher QOL score.

In our study, comorbidity is common among SCS. Nearly 76.7% of subjects had at least one comorbidity, and more than half of them had two or more comorbidities. Noticeably, nearly 50% of the SCS had digestive diseases, which was the most common comorbid condition in our samples. The potential adverse consequences of comorbidities pose a major challenge for the care of cancer survivors, and comorbidity has been shown to be an important prognostic factor for patients with cancer [16]. Furthermore, our results showed that these comorbidities impacted the QOL of SCS. Therefore, to better address the health needs of SCS, a more comprehensive understanding of relationships between comorbidities, cancer and QOL is needed. However, sequential provision of services (primary care interrupted by oncology care on an episodic basis) does not offer optimal care either for comorbidity or for cancer in the contemporary China [28]. Comprehensive cancer care must provide ongoing monitoring and management for the comorbidity of cancer survivors, which may not only prolong the survival [29], but also improve the QOL of cancer survivor.

Previous studies have demonstrated that healthy diet with enough vegetable and fruit intake was associated with better QOL of breast cancer survivors [23, 30]. Our research also reported similar findings that the SCS who ate more than 250 g of vegetables and some fruit every day were more likely to report higher overall QOL than those who did not engage in these healthy behaviors, indicating the benefits of consuming plenty of fruit and vegetable on QOL. Recent studies have suggested that increasing fruit and vegetable consumption can have beneficial effects on reducing recurrence or increasing survival for cancer survivors [31, 32]. Consistent with the 2010 Dietary Guidelines for Americans, cancer survivors should be encouraged to consume at least 2 to 3 cups of vegetables and 1.5 to 2 cups of fruits each day because of their health benefits [33]. Since it is difficult to identify which compounds of vegetables and fruits is beneficial the most, the reasonable advice is to consume plenty of various colorful vegetables and fruits daily.

Moreover, epidemiological studies have shown that high intakes of fruit and vegetables were associated with a lower risk of chronic diseases; particularly, cardiovascular disease [34, 35] and type 2 diabetes [36]. Consumption of vegetables and fruits impact not only cancer but also its commodities. Therefore, it is crucial to understand the importance of fruit and vegetable consumption, for comprehensive management program among SCS with comorbidities. However, changing behavioral risk factors and maintaining a healthy lifestyle were not easy for cancer survivors. Comprehensive chronic disease management models may be particularly appropriate in this regard, it should emphasize the continuity of long-term care, multidisciplinary cooperation, and specially, a relationship between patient and provider in which the patient was empowered and takes an active role in their ongoing care [37, 38], such as eating more vegetables and fruit.

The study has limitations that should be mentioned. First, all participants were the member of the SCRC, even though the SCRC recruited these members from community and hospitals using extensive recruitment channels. We acknowledge that selection bias may affect the study results. Second, information for only few clinical indicators was collected. Some important clinical indicators were not included, such as cancer stage, cancer metastatic and cancer recurrence, which could potentially confound the impact of comorbidity and consumption of fruit and vegetable on QOL. Examining the association between cancer clinical indicators and QOL will be the focus of our future study. Thirdly, the validity of self-reported comorbidity could be questionable [39], however, in our study we made it clear to the respondents that the comorbidity must be a clinical diagnosis. Given the cross-sectional nature of the study, we cannot deduce causal relationships between comorbidity, consumption of fruit and vegetable, and QOL of SCS, but merely describe probable associations. Further and different studies are needed to explore these kinds of associations.

## Conclusion

The comorbidities are common health problems among SCS and have significantly negative influence on QOL. High consumption of vegetables and fruit is positively associated with QOL of SCS. These findings warrant the comprehensive cares for SCS. A multidisciplinary team approach and a variety of delivery systems are needed to address the medical, psychosocial, and lifestyle components for enriching patient-centered care.

## Abbreviations

EORTC QLQ-C30: European Organization for Research and Treatment quality of life version 3 questionnaire
QOL: Quality of life
SCS: Stomach cancer survivors
